# Microstructural changes along the cingulum in young adolescents with psychotic experiences: An along‐tract analysis

**DOI:** 10.1111/ejn.15806

**Published:** 2022-09-12

**Authors:** Darren William Roddy, Elena Roman, Anurag Nasa, Areej Gazzaz, Ahmed Zainy, Tom Burke, Lorna Staines, Ian Kelleher, Aisling O'Neill, Mary Clarke, Erik O'Hanlon, Mary Cannon

**Affiliations:** ^1^ Department of Psychiatry Royal College of Surgeons in Ireland Dublin Ireland; ^2^ Trinity College Institute of Neuroscience, Lloyd Building Trinity College Dublin Dublin Ireland

**Keywords:** along‐tract analysis, cingulum, psychosis, psychotic experiences, tractography

## Abstract

Psychotic experiences (PEs) such as hallucinations and delusions are common among young people without psychiatric diagnoses and are associated with connectivity and white matter abnormalities, particularly in the limbic system. Using diffusion magnetic resonance imaging (MRI) in adolescents with reported PEs and matched controls, we examined the cingulum white matter tract along its length rather than as the usually reported single indivisible structure. Complex regional differences in diffusion metrics were found along the bundle at key loci following Bonferroni significance adjustment (*p* < .00013) with moderate to large effect sizes (.11–.76) throughout all significant subsegments. In this prospective community‐based cohort of school‐age children, these findings suggest that white matter alterations in the limbic system may be more common in the general non‐clinical adolescent population than previously thought. Such white matter alternations may only be uncovered using a similar more granular along‐tract analysis of white matter tracts.

## Graphical abstract

Psychotic experiences are common in young people without psychiatric diagnoses and may be associated with limbic white matter abnormalities. Complex regional differences (*p* < .00013) in diffusion metrics were found in key subsegments along the cingulum bundle in young adolescents aged 11–13 with psychotic experiences compared with age and sex matched controls. These white matter alternations were uncovered by examining the cingulum along its length (along‐tract analysis), rather than examining the tract as larger indivisible sections.

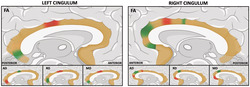

**Abbreviations**
ACC: anterior cingulate cortex
AD: axial diffusivity
ANVOCA: analyses of covariance
CSD: constrained spherical deconvolution
CSF: cerebrospinal fluid
dIPFC: dorsolateral prefrontal cortex
DWI: diffusion‐weighted imaging
eTIV: estimate of total intracranial volume
FA: fractional anisotropy
FDR: false discovery rate
ICC: interclass correlation coefficient
MD: mean diffusivity
mPFC: medial prefrontal cortex
MRI: magnetic resonance imaging
PCC: posterior cingulate cortex
PE: psychotic experiences
RD: radial diffusivity

## 1 INTRODUCTION

Psychosis, a common feature of many psychiatric, neurological and medical conditions, incorporates core symptoms of reality testing including delusions, hallucinations and thought disorders (American Psychiatric Association, 2013). Recently, the existence of an extended psychosis phenotype has been proposed suggesting that psychosis exists as a continuum, with varying degrees of reality testing (Van Os et al., 2009). Clinical cohorts, such as those with schizophrenia, lie at the extreme end of this continuum with most individuals experiencing limited psychotic experiences (PEs) and less fragmented reality testing. While the prevalence of diagnosable psychotic disorders is low, a significant number of people (global average 12.5%; ranging up to 45% in some countries) will experience at least one psychotic symptom in their lifetime (Andrade & Wang, 2012).

PEs, such as hallucinations or delusions, have been shown to be particularly common among young people (Kelleher, Connor, et al., 2012). Children or adolescents that are younger (9–12 years) have a higher prevalence of PEs compared with older (13–18 years) adolescents (Healy et al., 2019). Although most young people who describe PEs in early life do not develop a later psychiatric illness (Bennett et al., 2019), these individuals are at increased risk for a later diagnosis of a psychotic disorder such as schizophrenia (Poulton et al., 2000) as well other diagnosable psychiatric conditions such as depression and anxiety (Kelleher et al., 2013). Such individuals are also at increased risk of suicidal behaviour (Kelleher, Lynch, et al., 2012), poorer socio‐occupational function (Armando et al., 2010) and neurocognitive deficits (Blanchard et al., 2010). Further neurobiological research in these vulnerable young people may aid our understanding of how psychosis develops.

In recent years, evidence has emerged, which suggests that psychotic disorders and PEs are associated with connectivity and white matter abnormalities throughout the brain, particularly in the limbic system (Hegarty et al., 2019; O’Neill et al., 2020). Diffusion‐weighted imaging (DWI) is an MRI technique that models brain tracts by analysing the water diffusion properties of white matter. Recent advances in tractography, a process of reconstructing white matter tracts, allow for three‐dimensional visualisation and ‘virtual dissection’ of specific neuroanatomically defined white matter areas (Jeurissen et al., 2011; Roddy et al., 2018). Macrostructural (volume and length) and microstructural metrics can be examined in these reconstructed tracts. Microstructural metrics including axial diffusivity (AD), radial diffusivity (RD), mean diffusivity (MD) and fractional anisotropy (FA) are various measures of the diffusivity of water in the tract (Mori & Aggarwal, 2014) and can be broadly interpreted as proxies for white matter properties such as maturation, demyelination, membrane density and neuronal integrity, respectively (Alexander et al., 2011). Further recent developments, such as along‐tract analysis, allow a voxel‐specific analysis along a specific tract (Colby et al., 2012) permitting examination of microstructural metrics at the most granular level along the length of a tract.

**FIGURE 1:**
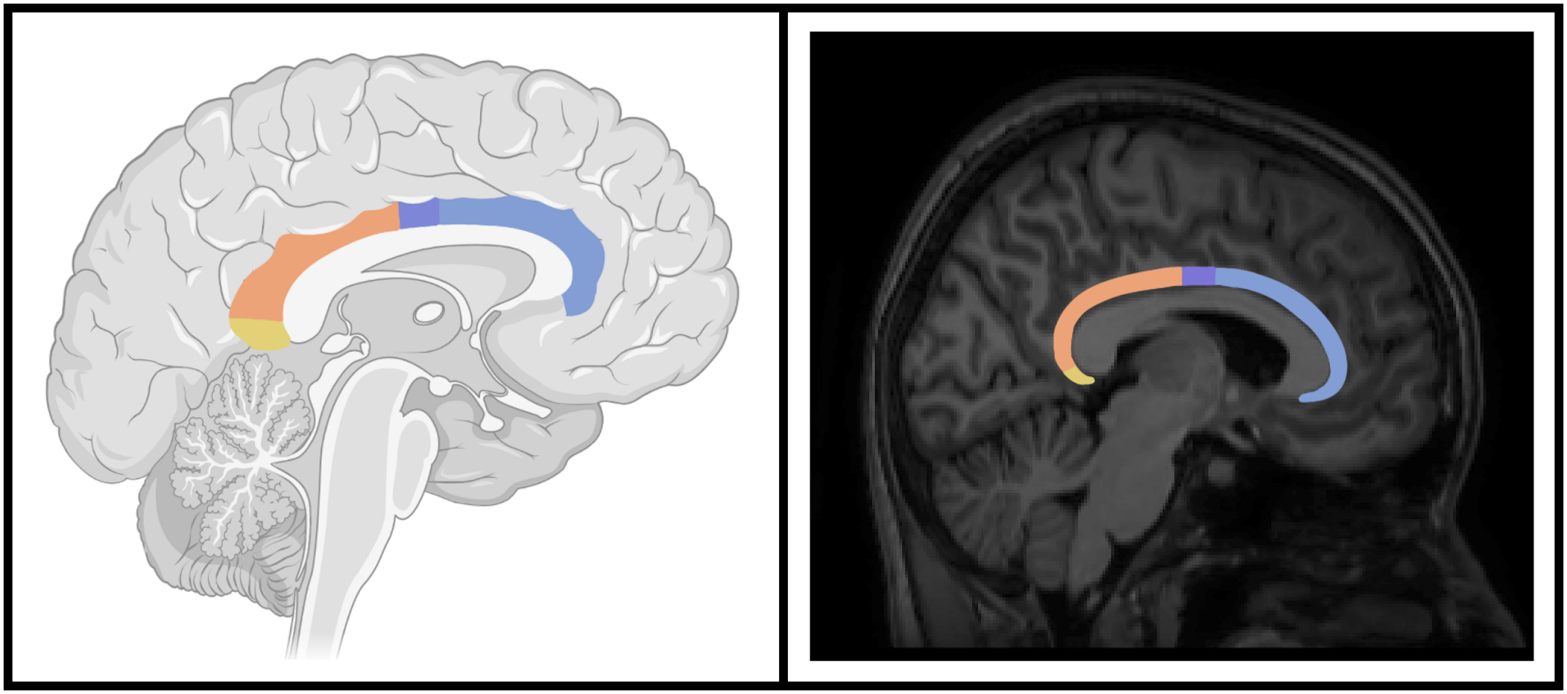
Cingulum bundle and diffusion sections. The left image shows a stylised cingulum bundle dorsal to the corpus callosum, lying within the grey matter of the cingulated cortex. The right image is a midline sagittal T1 image showing the right hemisphere with the cingulum bundle superimposed. Each cingulum has four sections: blue, subgenual; purple, body; orange, retrosplenial; yellow, parahippocampal.

**FIGURE 2:**
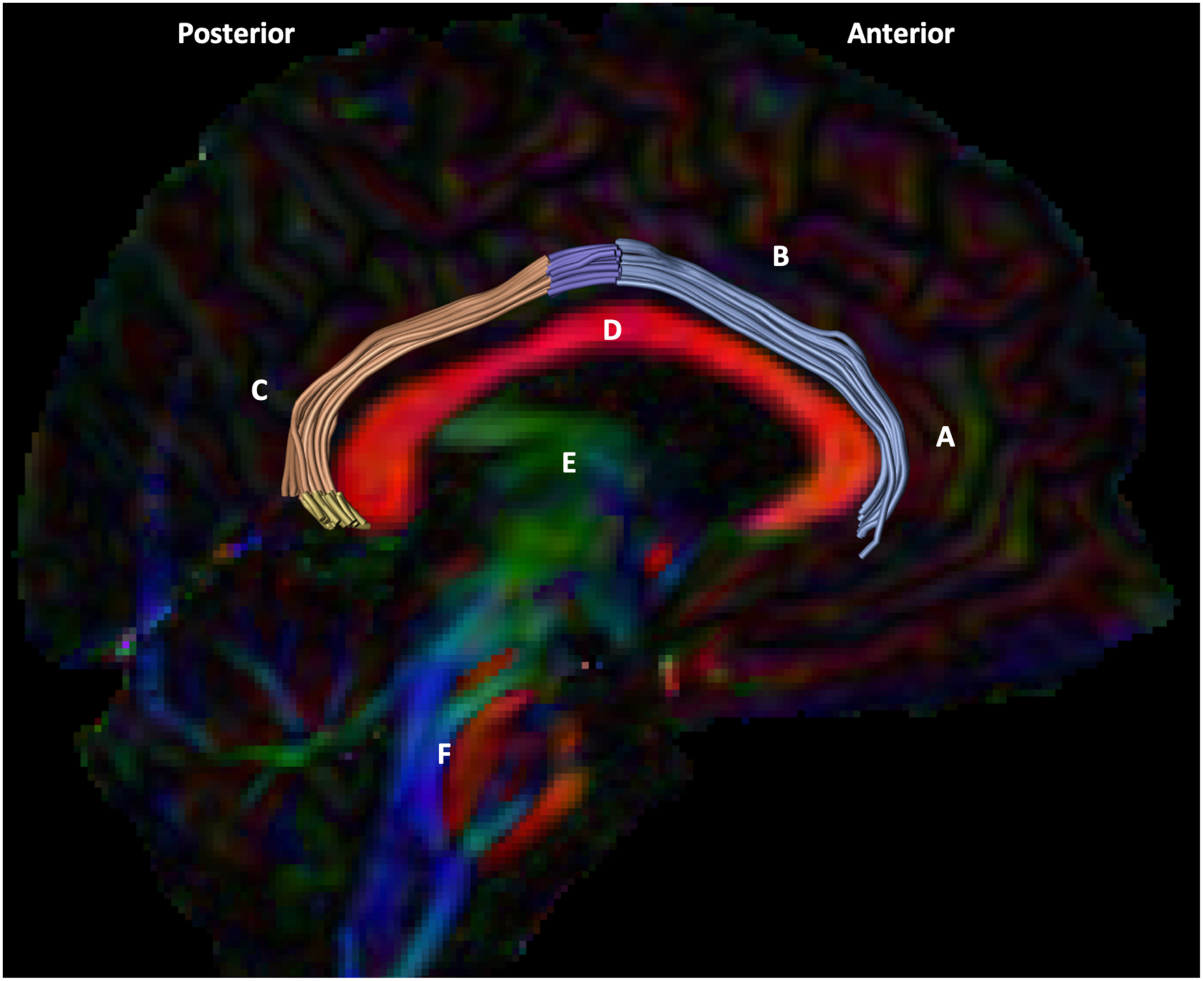
Cingulum in situ. Left cingulum with four sections; subgenual (blue); body (purple); retrosplenial (orange); parahippocampal (yellow) of a 16 year old male (control) on a midline sagittal first eigenvector fractional anisotropy (FEFA) diffusion map. Relative anatomical structures: medial prefrontal cortex (a); anterior cingulate cortex (b); retrosplenial cortex (c); corpus callosum (d); brainstem (e)

**FIGURE 3:**
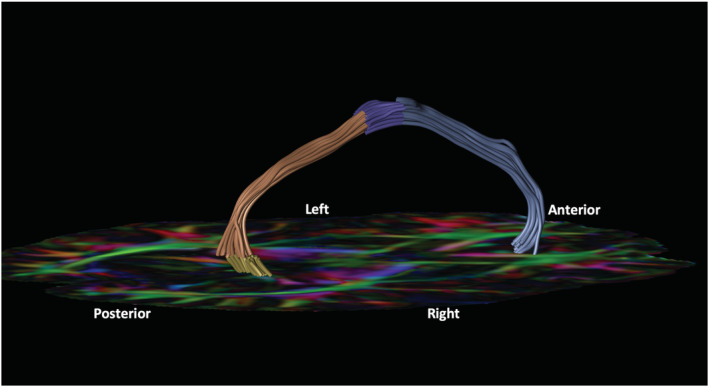
Four sections of the cingulum. Right cingulum of a 17 year old female (control). The cingulum is reconstructed from four sections: subgenual (blue); body (purple); retrosplenial (orange); parahippocampal (yellow); on an axial first eigenvector fractional anisotropy (FEFA) diffusion map at the level of the anterior commissure.

The hippocampus, a key temporal structure involved in memory and spatial awareness, has been found to be disturbed in many psychiatric conditions (Nolan et al., 2020; Roddy & O'Keane, 2019) including early psychosis (Calvo et al., 2020). Major connections from the hippocampus to the rest of the limbic system include the fornix, dorsal hippocampal commissure (Nasa et al., 2021) and cingulum. This bundle extends from the posterior hippocampus over the corpus callosum to the prefrontal cortex. The cingulum connects the overlying frontal, parietal, cingulate and temporal regions (Bubb et al., 2018) as it forms part of the traditional Papez circuit (Weininger et al., 2019), underpinning its role in functions such as memory, attention and emotional processing (Figures 1 and 2). Although often examined in neuroimaging as a single uniform bundle, stretching from the hippocampus to the prefrontal cortex, the cingulum constitutes numerous short and long cortico‐cortical association fibres linking overlying regions along its path (Vogt & Paxinos, 2014). These cytoarchitecturally and topographically distinct connections connect adjacent limbic structures, such that relatively few fibres span the entirety of the cingulum tract (Bubb et al., 2018). Evidence from tracing studies suggests that the cingulum may be divided into at least four functional ‘sections’ (Bubb et al., 2018), allowing more anatomically accurate and localised investigation using DWI. Using standard anatomical landmarks, the cingulum has been subdivided into four stable sections from anterior to posterior (Figures 1 and 3); a subgenual section (underneath and in front of the corpus callosum); a main body section (along the dorsum of the corpus callosum); a retrosplenial section (curving around the back of the corpus callosum); and finally the parahippocampal section (easing down towards the hippocampal circuitry) (Jones et al., 2013). Each section corresponds to a unique topographic fibre arrangement, allowing more precise localisation of cingulum changes in neuropsychological disorders using DWI.

The majority of studies in psychotic disorders have considered the cingulum as a single structure, rather than investigating the tract at a more localised level. Notwithstanding, whole cingulum bundle differences are widely reported in patients with schizophrenia, with patients showing smaller cinguli with reduced FA (Kubicki et al., 2003). Differences have been found at various levels of cingulum subdivision in schizophrenia when considering a simple anterior–posterior level (Fujiwara et al., 2007), a section level (Whitford et al., 2014) and along each voxel of the tract (Abdul‐Rahman et al., 2011). However, the evidence is limited and mixed when examining cingulum sections in high‐risk individuals on the psychosis continuum without a psychiatric diagnosis (Epstein et al., 2014; Fitzsimmons et al., 2020; Peters et al., 2008). For those individuals with mild or fleeting symptoms, for example, those at the psychosis continuum threshold, cingulum changes may only be discovered through more localised, rather than unitary/sectional, investigations. To date, no study has applied a more detailed, granular, along‐tract analysis to investigating the cingulum in these individuals.

We hypothesise that differences exist at the level of the four cingulum sections in young people with PEs compared with matched controls. To this end, we assessed a cohort of adolescents with PEs from the general school‐going population (who had not yet presented to services) and healthy controls using high‐resolution DWI and reconstructed four sections of the cingulum. We further hypothesise that differences exist along subsegments of each section in PEs. With the use of along‐tract analysis, we divided each section into subsegments to determine if any deeper, subtler differences were revealed.

## 2 METHODS

### 2.1 Participants

This study involves a prospective case–control sample of adolescents aged 11–13 years, which included 25 young people with PEs (as rated by a consensus panel of clinicians with expertise in psychosis) and 25 matched for age, sex and handedness young people without PEs. Participants were medically well children prospectively recruited from schools in Ireland as part of the *Adolescent Brain Development Study* (Kelleher, Harley, et al., 2011). Initially, 212 adolescents were interviewed for the study. All were invited for scanning; however, only 100 agreed and consented to MRI scanning. Of the 100 respondents, 25 reported PEs and the remaining 75 participants reported no PEs. From the 75 reporting no PEs, 25 were selected to match the age, sex and handedness of the other 25 adolescents with PEs. All participants had no contraindications to MRI and were psychotropic medication and illicit substance naïve. All participants underwent a diagnostic clinical interview with trained raters (Kelleher, Murtagh, et al., 2011). Further recruitment details are provided elsewhere (Kelleher, Harley, et al., 2011). Psychotic symptoms were assessed using the psychosis section of the Schedule for Affective Disorders and Schizophrenia for School‐aged Children (K‐SADS). If a participant elicited a positive response to a screening question, further details were determined using the *SOCRATES* template (https://epubs.rcsi.ie/psychart/19/). This allowed systematic and comprehensive documentation of the perceptual and thought content abnormalities. Each participant was discussed at a consensus meeting involving three mental health professionals with expertise in psychosis (MC, IK, MH), where a decision was reached on whether the young person reported a ‘definite’ psychotic symptom. Auditory verbal hallucinations were present in >90% of those reporting symptoms relating to PE. Otherwise, these were medically well children.

Ethical approval was obtained from the Medical Research Ethics Committee, Beaumont Hospital, Dublin. Written parental consent and participant assent were obtained prior to the study.

### 2.2 MRI data acquisition

All data were acquired on a Philips Intera Achieva 3.0 Tesla‐MR system (Best, Netherlands) with an 8‐channel head coil in Trinity College Institute of Neuroscience, Dublin. Whole‐brain, high angular resolution diffusion imaging (HARDI) was acquired using a spin‐echo echo‐planar imaging pulse sequence (TE = 52 ms, TR = 11,260 ms, flip angle alpha = 90°), FOV 224 mm, 60 axial slices 1.75 × 1.75 mm^2^ in‐plane resolution, slice thickness 2 mm, *b*‐value = 1500 s mm^−2^ in 61 non‐collinear gradient directions. A non‐diffusion‐weighted volume was acquired for image registration and motion correction; 180 axial high‐resolution T1‐weighted anatomical images (T1W‐IR1150 sequence, TE = 3.8 ms, TR = 8.4 ms, FOV 230 mm, .898 × .898 mm^2^ in‐plane resolution, slice thickness .9 mm, flip angle alpha = 8°) were also acquired.

### 2.3 MR data analyses

Pre‐processing and tractography analyses were performed using ExploreDTI 4.8.5 (http://www.ExploreDTI.com) (Leemans et al., 2009). Subject motion, eddy current and echo‐planar imaging distortion were corrected (Leemans & Jones, 2009), and affine co‐registration of the diffusion data to non‐distorted T1 images was performed. Constrained spherical deconvolution (CSD)‐based fibre tractography (Jeurissen et al., 2011), demonstrating an enhanced performance in regions of complex white matter architecture (McGrath et al., 2013; Wilkins et al., 2015), was used to generate whole‐brain tracts. An estimate of total intracranial volume (eTIV) for covariate analysis was generated using the T1 images and Freesurfer 6.0.

### 2.4 Cingulum tractography

Using carefully deployed ‘AND’ and ‘NOT’ gates, the left and right cingulum sections in every subject were reconstructed (to generate a total of 200 sections across 50 subjects). Using Boolean logic, ‘AND’ gates capture all streamlines traversing the plane within the gate boundaries, whereas ‘NOT’ gates remove all streamlines traversing the plane within the gate boundaries. Combinations of ‘AND’ and ‘NOT’ gates can be used to capture specific tracts from the previously rendered whole‐brain tracts.

Each cingulum was isolated by two independent trained raters (ER, AN) using the Jones et al. (2013) protocol for dividing the cingulum into four discrete sections; subgenual, body, retrosplenial and parahippocampal regions (Jones et al., 2013) (Figure 1). The original Jones et al. (2013) protocol generated three discrete regions (subgenual, retrosplenial and parahippocampal), with a 10‐slice gap along the body between the subgenual and retrosplenial. Our protocol adds a fourth section in the middle of the cingulum body to fill in the 10‐slice void that we term ‘body’. Therefore, our protocol investigates the cingulum along 4 contiguous parts with no gaps, running posteriorly from underneath the genu of the corpus callosum to underneath/behind the splenium of the corpus callosum.

Initially, the entire cingulum was visualised by an ‘AND’ gate around the mid‐cingulum above the corpus callosum to assist in the proper anatomical placement of the section gates, as well as inspection of the general condition of the cingulum. This was deleted as necessary. First, two ‘AND’ gates were placed at five slices posterior and five slices anterior to the rostrocaudal midpoint of the cingulum body, that is, the midpoint between the back of the curve of the genu and the front of the splenium. This generated what Jones et al. (2013) term the standard cingulum—a representation of the cingulum along its length. This 10‐slice section (described as ‘body’ by us) was isolated using the ‘segment tool’ in ExploreDTI, retaining the fibres between the two ‘AND’ gates 10 slices apart and removing streamlines external to this. The subgenual region was visualised using the anterior ‘AND’ gate of the body section and a second AND gate placed on the third coronal slice caudal to the most anterior part of the genu, that is, its anterior limit. This was isolated into a separate section using the ‘segment tool’ in ExploreDTI. The retrosplenial region was visualised using the posterior ‘AND’ gate of the body section and a second AND gate placed on the most ventral plane of the splenium (a horizontal section three or four slices (∼6 mm) above the splenium base) and similarly isolated with the ‘segment tool’. Finally, the parahippocampal region was visualised using the second AND gate of the retrosplenial section, and another AND gate placed four slices below this along the cingulum tract. This section was then isolated using the ‘segment tool’ in ExploreDTI.

Each section was inspected visually, and spurious streamlines were removed consistently according to a predefined protocol. This protocol involved using ‘NOT’ gates to remove these non‐cingulum streamlines. Criteria for exclusion included streamlines deviating from the known shapes of each cingulum section and streamlines consistent with known adjacent tracts such as the corpus callosum, superior longitudinal fasciculus, fornix and thalamic projections.

An initial interclass correlation coefficient ICC) was calculated on the initial 20% (*n* = 40) of the tracts to examine interrater reliability. Raters' methods adjusted iteratively if required until the ICC between raters was greater than .9. A third rater (DWR) randomly reconstructed 10 sections that were crosschecked with the other raters to further ensure the reliability of the method.

Volumes and diffusion tensor metrics were calculated for each tract section using the ‘info of tract’ plugin in ExploreDTI. This plugin calculates the tract average AD, RD, MD, FA, tract lengths and volumes of each section. Tract resampling (using the ‘uniform tract resampling’ plugging in ExploreDTI) was performed to localise diffusion metric variations along the length of each cingulum section (Colby et al., 2012; O’Hanlon et al., 2015). Each section was divided into equal subsegments corresponding to the maximum number of voxels along the section in question. The maximum number of voxels along each section was calculated by dividing the mean tract length by the maximum diffusion voxel dimension (in our case, the 2 mm slice thickness). Mean tract lengths were 58.7 mm (subgenual), 10.4 mm (body), 20.6 mm (retrosplenial) and 6.3 mm (parahippocampal) resulting in 29, 5, 10 and 3 subsections in each section, respectively. This allowed examination of the maximum number of discrete subsegments along the length of each section (Dooley et al., 2020; O’Hanlon et al., 2015; Roddy et al., 2018). Diffusion metrics were extracted at each of these evenly spaced subsegments allowing point‐wise correspondence across subjects.

### 2.5 Statistical analyses

Macroscopic and microscopic data from the cingula sections were inspected visually and then exported to IBM SPSS Statistics 26. One‐way between‐group analyses of covariance (ANCOVAs) compared mean differences between PE and control groups separately for the four diffusion metrics and volume in each section on both sides. Age, sex and estimated total intracranial volume (eTIV) were controlled as covariates. Bonferroni corrections for multiple comparisons adjusted the threshold for significance for cingulum sections to *p* < .002 and volume to *p* < .0063.

Similarly, one‐way ANCOVA compared mean differences between PE and control groups for diffusion metrics of each subsegment in each section on both sides. Age, sex and eTIV we similarly controlled as covariates as in the previous section analysis. Both Bonferroni and false discovery rate (FDR) corrections for multiple comparisons were performed. The most strict significance threshold was chosen (Bonferroni) with an adjusted significance for the cingulum subsegments of *p* < .00013. Effect size was denoted by partial eta^2^ (.01 = low, .09 = moderate, .25 = large).

## 3 RESULTS

No differences between groups for age, sex or handedness were found (Table 1).

**TABLE 1:** Demographics

	PE (*n* = 25)	Controls (*n* = 25)	*p* value
Mean age (years)	13.5 (1.26)	13.36 (1.15)	.642
Sex (male)	8 (32%)	10 (40%)	.765
Handedness (right)	23 (92%)	24 (96%)	.552

*Note*: Demographic data of 25 adolescents with PEs and 25 controls.

Abbreviations: PE, adolescents with psychotic experiences; *SD*, standard deviation.

### 3.1 Cingulum sections

Following Bonferroni significance adjustment (*p* < .0063), no volume differences were found between groups for any cingulum section. However, the left subgenual (*p* = .014), left body (*p* = .033) and left (*p* = .032) and right (*p* = .011) parahippocampal section volumes were smaller in the PE group at a trend level when uncorrected for multiple comparisons (Table 2). Similarly, no diffusion metric differences were found between groups following Bonferroni correction (*p* < .002) for any cingulum section. However, the right retrosplenial section exhibited a larger RD at the trend level (*p* = .005).

**TABLE 2:** Cingulum section results

	Volume (mm^3^)	AD	RD	MD	FA
Left					
Subgenual	.014	.573	.68	.713	.614
Body	.033	.525	.157	.197	.295
Retrosplenial	.19	.607	.58	.573	.744
Parahippocampal	.032	.808	.746	.757	.901
Right					
Subgenual	.103	.812	.51	1	.79
Body	.098	.772	.247	.279	.163
Retrosplenial	.694	.569	.005	.071	.103
Parahippocampal	.011	.626	.46	.704	.575
Bonferroni	.00625	.002	.002	.002	.002

*Note*: Sectional analysis of the left and right cingulum following ANCOVA (correcting for age, sex and eTIV) between PEs and controls. All results are significant at following Bonferroni correction detailed at end of the table.

Abbreviations: AD, axial diffusivity; ANCOVA, analysis of covariance; eTIV, estimated total intracranial volume; FA, fractional anisotropy; MD, mean diffusivity; PE, adolescents with psychotic experiences; RD, radial diffusivity.

### 3.2 Cingulum subsegments

**FIGURE 4:**
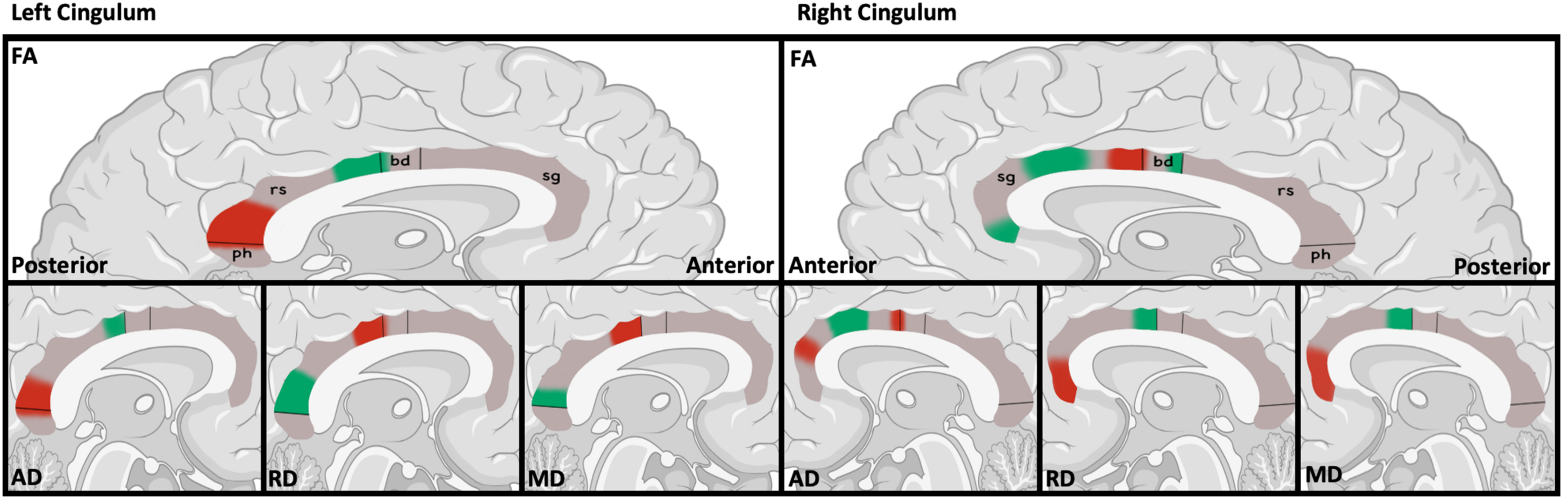
FA changes along the cingulum in young adolescents with psychotic experiences. Stylised left and right cingulum showing position and direction of FA, AD, RD and MD changes along the bundle. Green denotes diffusion metric increase; red denotes a decrease. FA increases are shown corresponding to the overlying left medial prefrontal and right caudal anterior cingulate cortices (see Figure 5). FA decreases are shown corresponding to the overlying right anterior cingulate and left retrosplenial cortices*.* AD, axial diffusivity; bd, body; FA, fractional anisotropy; MD, median diffusivity; ph, parahippocampal; RD, radial diffusivity; rs, retrosplenial; sg, subgenual

Following Bonferroni significance adjustment (*p* < .00013), differences in diffusion metrics were found along continuous subsegments of the left retrosplenial/body (Table 3, Figure 4). These can be grouped into five broad continuous subsegments of differences. The left cingulum subsegments revealed two broad patterns of differences: (1) The caudal body and rostral retrosplenial subsegments showed increased AD and FA with decreased MD and RD, whereas (2) the left caudal retrosplenial rostral/parahippocampal subsegments showed an opposite pattern of decreased AD and FA with increased MD and RD. The right cingulum subsegments revealed three broad patterns of differences: (3) the right subgenual showed rostral increased FA with decreased MD and RD; (4) the caudal‐mid subgenual showed increased AD and FA; and (5) caudal subgenual/rostral body increased MD and RD and decreased AD and FA. Moderate to large effect sizes (.11–.76) were found throughout all significant subsegments. Occasional differences were found for isolated single subsegments in the left retrosplenial and bilateral body. The left subgenual and right retrosplenial sections showed no differences in any diffusion metrics.

**TABLE 3:** Cingulum along‐tract analysis results

	Left				Right			
Section	AD	RD	MD	FA	AD	RD	MD	FA
Subgenual 1	NS	NS	NS*	NS	↑(.16)	↓(.61)	↓(.35)	↑(.49)
Subgenual 2	NS	NS	NS*	NS	NS*	↓(.60)	↓(.42)	↑(.39)
Subgenual 3	NS	NS	NS	NS	NS	↓(.55)	↓(.41)	↑(.29)
Subgenual 4	NS	NS	NS	NS	NS	↓(.50)	↓(.40)	↑(.20)
Subgenual 5	NS	NS	NS	NS	NS	↓(.44)	↓(.35)	↑(.13)
Subgenual 6	NS	NS	NS	NS	NS	↓(.35)	↓(.30)	NS*
Subgenual 7	NS	NS	NS	NS	NS	↓(.24)	↓(.23)	NS
Subgenual 8	NS	NS	NS	NS	NS	NS*	↓(.19)	NS
Subgenual 9	NS	NS	NS	NS	↓(.18)	NS	↓(.15)	NS*
Subgenual 10	NS	NS	NS	NS	↓(.19)	NS	↓(.12)	↓(.13)
Subgenual 11	NS	NS	NS	NS	↓(.13)	NS	NS*	NS
Subgenual 12	NS	NS	NS	NS	NS	NS	NS	NS
Subgenual 13	NS	NS	NS	NS	NS	NS	NS	NS
Subgenual 14	NS	NS	NS	NS	NS	NS	NS	NS
Subgenual 15	NS	NS	NS	NS	NS	NS	NS	NS
Subgenual 16	NS	NS	NS	NS	NS	NS	NS	↑(.11)
Subgenual 17	NS	NS	NS	NS	↑(.17)	NS	NS	↑(.23)
Subgenual 18	NS	NS	NS	NS	↑(.16)	NS	NS	↑(.21)
Subgenual 19	NS	NS	NS	NS	↑(.21)	NS	NS	↑(.30)
Subgenual 20	NS	NS	NS	NS	↑(.24)	NS	NS	↑(.35)
Subgenual 21	NS	NS	NS	NS	↑(.24)	NS	NS	↑(.23)
Subgenual 22	NS	NS	NS	NS	↑(.18)	NS	NS	↑(.17)
Subgenual 23	NS	NS	NS	NS	↑(.15)	NS	NS*	↑(.11)
Subgenual 24	NS	NS	NS	NS	NS	NS	↑(.17)	NS
Subgenual 25	NS	NS	NS	NS*	NS	↑(.19)	↑(.12)	NS
Subgenual 26	NS	NS	NS	NS*	NS	↑(.34)	↑(.22)	↓(.13)
Subgenual 27	NS	NS	NS	NS*	NS	↑(.29)	↑(.16)	↓(.18)
Subgenual 28	NS	NS	NS	NS	↓(.18)	↑(.42)	↑(.19)	↓(.32)
Subgenual 29	NS	NS	NS	NS	↓(.23)	↑(.43)	↑(.22)	↓(.34)
Body 1	NS	NS	↓(.10)	NS	↓(.14)	NS	NS	↓(.13)
Body 2	NS	NS*	NS	NS*	NS	NS	NS	NS
Body 3	NS	NS	NS	NS	NS	NS	NS	NS
Body 4	NS	NS	NS	NS	NS*	NS*	NS	↑(.17)
Body 5	NS*	↓(.23)	NS	↑(.25)	↑(.17)	NS*	NS	↑(.25)
Retrosplenial 1	↑(.35)	↓(.70)	↓(.47)	↑(.67)	NS	NS*	NS	NS
Retrosplenial 2	↑(.23)	↓(.68)	↓(.51)	↑(.58)	NS	NS	NS	NS
Retrosplenial 3	↑(.17)	↓(.60)	↓(.45)	↑(.50)	NS	NS	NS	NS
Retrosplenial 4	NS	↓(.47)	↓(.47)	↑(.34)	NS	NS	NS*	NS
Retrosplenial 5	NS	↓(.22)	↓(.29)	NS*	NS	NS	NS	NS
Retrosplenial 6	NS	NS	NS*	NS	NS	NS	NS	NS
Retrosplenial 7	NS	NS	↓(.13)	NS	NS	NS	NS*	NS
Retrosplenial 8	NS	NS	NS*	NS	NS	NS	NS	NS
Retrosplenial 9	NS*	NS	NS	NS	NS	NS	NS	NS
Retrosplenial 10	↓(.16)	NS	NS	↓(.15)	NS	NS	NS	NS
Retrosplenial 11	NS	NS	NS	NS	NS	NS*	NS*	NS
Retrosplenial 12	NS	NS	NS	NS	NS	NS	NS	NS
Retrosplenial 13	NS	NS	NS	NS	NS	NS	NS	NS
Retrosplenial 14	NS	NS	NS	NS	NS	NS*	NS*	NS
Retrosplenial 15	NS	NS	NS	NS	NS	NS*	NS	NS*
Retrosplenial 16	NS	NS	NS	NS	NS	NS*	NS	NS*
Retrosplenial 17	NS	NS	NS	NS	NS*	NS*	NS	NS*
Retrosplenial 18	NS	NS	NS	NS	NS*	NS*	NS	NS*
Retrosplenial 19	NS	↑(.22)	NS	↓(.14)	NS*	NS*	NS	NS*
Retrosplenial 20	NS	↑(.18)	NS	↓(.15)	NS	NS	NS	NS
Retrosplenial 21	NS	↑(.17)	NS	↓(.14)	NS	NS	NS	NS
Retrosplenial 22	↓(.16)	↑(.33)	NS*	↓(.31)	NS	NS	NS	NS
Retrosplenial 23	↓(.43)	↑(.61)	↑(.17)	↓(.64)	NS	NS	NS	NS
Retrosplenial 24	↓(.52)	↑(.75)	↑(.39)	↓(.73)	NS	NS	NS	NS
Retrosplenial 25	↓(.53)	↑(.76)	↑(.45)	↓(.76)	NS	NS	NS	NS
Parahippocampal 1	↓(.15)	NS*	NS	↓(.17)	NS	NS	NS	NS
Parahippocampal 2	NS	NS	NS	NS	NS	NS	NS	NS
Parahippocampal 3	NS*	NS*	NS	NS*	NS	NS	NS	NS*

*Note*: Along‐tract analysis of the left and right cingulum following ANCOVA (correcting for age, sex and eTIV) between PEs and controls. All results are significant at *p* < .00013 following Bonferroni correction. Arrows and brackets indicate directions of difference in PE group and effect sizes (partial eta^2^ [.01 = low, .09 = moderate, .25 = large]).

Abbreviations: AD, axial diffusivity; ANCOVA, analysis of covariance; eTIV, estimated total intracranial volume; FA, fractional anisotropy; MD, mean diffusivity; PE, adolescents with psychotic experiences; RD, radial diffusivity.

* Indicates significance at uncorrected *p* < .05.

## 4 DISCUSSION

Using a combination of sectional and along tract analysis, this study examined the cingulum in a cohort of young adolescents with PEs compared with healthy controls. Although no differences were found at the level of cingulum sections, region‐specific clusters of microstructural change were found in the left retrosplenial and right subgenual areas using along‐tract analysis. This is the first study to use both section and along tract analysis in young adolescents with PEs, and the first study to show that along tract analysis may be useful in uncovering deeper, previously hidden, differences in the cingulum.

### 4.1 Sectional analyses show no differences in PEs

No diffusion differences between PEs and controls were found when each cingulum bundle was examined as four separate sections; however, trend RD increases were found in the right retrosplenial section (Table 2). Cingulum differences in schizophrenia have been found when examined as a single structure (Fujiwara et al., 2007; Kubicki et al., 2003) and when subdivided into two (Fujiwara et al., 2007; Takei et al., 2009), four (Abdul‐Rahman et al., 2011) or five sections (Whitford et al., 2014). Regionally specific disruptions have also been found using a voxel‐by‐voxel approach along the bundle in schizophrenia (Abdul‐Rahman et al., 2011). However, the evidence for cingulum differences along the psychosis continuum is more mixed. Decreased FA was found using whole‐brain approaches in genetic high‐risk individuals (Hoptman et al., 2008) and the anterior cingulum of clinical individuals using voxel‐based morphometry (Zhou et al., 2017). Differences have also been associated with positive prodromal psychotic symptoms in subjects with 22q11.2 deletion syndrome (Kates et al., 2015), and lower right whole cingulum FA has been found in schizotypal personality disorder (Hazlett et al., 2011). Interhemispheric connectivity differences have been found in the anterior and posterior cingulum bundle with psychotic‐like experiences in healthy individuals (Oestreich et al., 2019). Using tractography, lower cingulum FA was found in a recent study of clinical high‐risk adolescents (Fitzsimmons et al., 2020) while a larger study of healthy adolescents, early‐onset schizophrenia adolescents and clinical high‐risk adolescents found no differences between groups (Epstein et al., 2014). Our results are consistent with the only other tractography study to date dividing the bundle into sections (two), showing no differences found in either the dorsal or anterior cingulum between ultra high‐risk individuals, new schizophrenia patients and controls (Peters et al., 2008). Taken together, these heterogeneous findings suggest that techniques examining the bundle at a more granular level may reveal the more subtle differences present in an already subtle phenotype.

### 4.2 Robust subsegmental changes underlying key cortical areas in PEs

**FIGURE 5:**
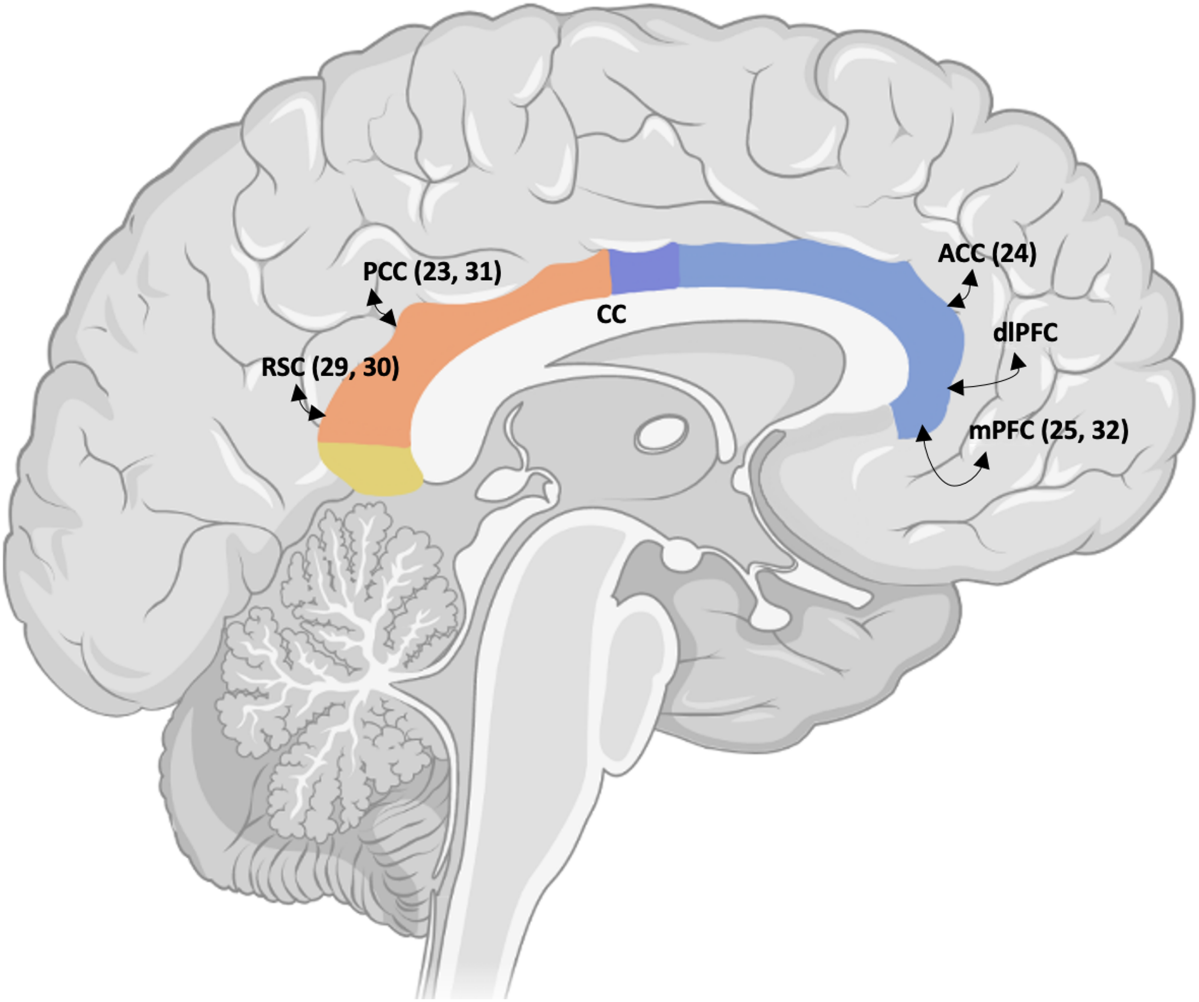
Connectivity with overlying and adjacent regions along the cingulum bundle. Stylised midline sagittal image showing the right cingulum reconstructed from four sections: subgenual (blue); body (purple); retrosplenial (orange); parahippocampal (yellow). The approximate connections to and from the bundle are to the overlying cortical regions along its length. ACC, anterior cingulate cortex; dlPFC, dorsolateral prefrontal cortex; mPFC, medial prefrontal cortex; PCC, posterior cingulate cortex, RSC; retrosplenial cortex

Using along‐track analysis, complex multidirectional diffusion differences were found in the left retrosplenial and right subgenual regions. All findings were highly significant following multiple comparison corrections (*p* < .00013). Likewise, all significant findings demonstrated moderate to high effects sizes, with the left retrosplenial segments showing particularly high effects sizes (eta^2^ = highest, .76; average, .38). FA is a measure of the degree of anisotropy of diffusion along a bundle i.e. how ‘restricted’ the relative diffusion of the first eigenvector (diffusion along the bundle) is relative to the other two eigenvectors (diffusion across the bundle). Higher FA reflects increased diffusivity along the direction of the tract. Axonal diameter, fibre density and myelination are all known to influence FA (Mori & Aggarwal, 2014). However, complex crisscrossing fibres and extensive dendritic arborisation, as found in maturing brains, reduce diffusivity coherence and lower FA. As FA is calculated using the three eigenvectors across all dimensions, changes in FA are complex and highly dependent on factors influencing AD (the first eigenvector along the bundle direction), RD (the average of the remaining two ‘non‐bundle’ direction eigenvectors), MD (the average of all three eigenvectors across all directions) and the local microstructure of region. The cingulum is a complex structure connecting to the overlying cortical regions along its length (Figure 5). Our clusters of diffusion differences along the cingulum in adolescents with PEs topographically correspond with the overlying dorsolateral prefrontal cortex (dlPFC), medial prefrontal cortex (mPFC), posterior cingulate cortex (PCC), anterior cingulate cortex (ACC) and parietal cortex, which may, in part, explain deficits in neuropsychological function associated with these regions for people with PE compared with controls (Figure 5) (Carey et al., 2019, 2020; Coughlan et al., 2021; Kelleher et al., 2015).

### 4.3 Increased FA with increased AD underlying the right dlPFC

AD corresponds to the first diffusion eigenvector and may indicate axonal maturation but may also suggest smaller axonal calibre and reduced neuropil secondary to reduced dendritogenesis (Mori & Aggarwal, 2014). It is thought not to reflect myelin changes. Increased AD was found along the mid‐right subgenual area corresponding with cingulum connections to and from the right dlPFC.

The dlPFC is involved in executive functions including decision‐making, working memory, abstract reasoning and social cognition (Frangou et al., 2005; Stuss & Knight, 2013). Reduced right dlPFC activation during visuospatial working memory tasks has been demonstrated in patients with first‐episode psychosis (Jalbrzikowski et al., 2018). At‐risk mental state individuals also show reduced dlPFC activity in working memory tasks (Fusar‐Poli et al., 2010), with activity shown to be intermediate between controls and patients diagnosed with psychosis (Broome et al., 2009). Decreased right dlPFC activity as measured by lower oxygenated haemoglobin using functional near‐infrared spectroscopy has also been found in high‐risk and ultra‐high‐risk groups compared with controls (Aleksandrowicz et al., 2020). Our findings are suggestive of reduced neurogenesis support evidence of decreased activity of the maturing dlPFC as it connects with the cingulum complex (Table 3, Figure 4).

### 4.4 Increased FA with decreased RD (and MD) underlying the left caudal ACC and right mPFC

RD is a measure of average diffusivity perpendicular to the direction of the axonal fibres. Decreased RD reflects reduced axonal packing and myelination (Mori & Aggarwal, 2014). MD is a measure of the average diffusivity in all directions and is a somewhat non‐specific measure of neuropil or cerebrospinal fluid (CSF) infiltration (Mori & Aggarwal, 2014). Reduced MD and RD were found along the left rostral retrosplenial and right rostral subgenual areas in young adolescents with PEs (Table 3). This anatomical distribution corresponds with the overlying left caudal ACC and right mPFC (Figure 5). As RD differences show the largest effect size, reduced perpendicular diffusion is the major driver of FA increase, suggesting tightly packed or highly myelinated axons in these regions.

The medial PFC has reciprocal connections with the amygdala (emotional recognition and processing), hippocampus (memory) and temporal cortex (higher‐order sensory regions), making it important for social decision‐making and emotional processing (Stuss & Knight, 2013). Schizophrenia is associated with mPFC hyperactivity (Taylor et al., 2007). Subjects with ultra‐high risk also show increased mPFC activation (Shim et al., 2010), with those subsequently developing psychosis demonstrating increased activation and greater mPFC–midbrain connectivity compared with those who did not become psychotic. Failure to deactivate the mPFC during working memory tasks in those at clinical high risk for psychosis compared to familial high‐risk (Falkenberg et al., 2015) suggests that activity in this area may be associated with a unique vulnerability to psychosis. The mPFC shows hyperactivation and hyper‐connectivity in patients with schizophrenia and first‐degree relatives (Whitfield‐Gabrieli et al., 2009) and in those at ultra‐high risk also (Shim et al., 2010).

Auditory verbal hallucinations were present in the overwhelming majority of our young adolescents with PEs (>90%). Auditory hallucinations are hypothesised to occur due to conflicts between auditory processing and internal monitoring (Hugdahl, 2015). The most caudal (posterior) region of the ACC has shown activation with increased task interference in an auditory Stroop task (Haupt et al., 2009). Similarly, psychosis scores (including auditory hallucinations) were correlated with caudal ACC regional cerebral blood in the caudal ACC in drug‐free patients with schizophrenia (Lahti et al., 2006).

### 4.5 Decreased FA underlying the right ACC and left retrosplenial cortex

Increased MD & RD with decreased AD reflects reduced diffusivity along the posterior left retrosplenial and right subgenual sections in the PE group. Phenomena known to cause this pattern include reduced myelination, loose axonal packing and CSF/inflammatory infiltration (Alexander et al., 2011). This form of FA decrease was found underlying the left retrosplenial cortex and right ACC in young adolescents with PEs (Table 3). Our findings of reduced cingulum coherence may reflect findings of hypofunction in these overlying cortical regions in psychosis (Table 3, Figure 4).

The ACC has roles in self‐monitoring, social cognition and salience processing, each of which are functions perturbed in schizophrenia (Garavan et al., 1999). ACC hypoactivity has been associated with schizophrenia (Bersani et al., 2014) and people who are considered at‐risk (Lord et al., 2012). Reduced ACC cortical thickness and grey matter has also been observed in high‐risk individuals who go on to develop psychosis (Andreou & Borgwardt, 2020).

The retrosplenial cortex may have a role in mediating between perception and memory (Vann et al., 2009). It has been suggested that it may act as a conduit between egocentric (self‐centred) and allocentric (world‐centred) information (Miller et al., 2014). Weakened retrosplenial cortex connectivity has been observed in patients with schizophrenia (Bluhm et al., 2009), as well as decreased activity (Tendolkar et al., 2004), and reduced volume is associated with poor patient outcome in schizophrenia (Mitelman et al., 2005). Of note, the left posterior retrosplenial regions showed the highest *average* effect size in our study (partial eta^2^ = .38).

In total, our robust (high significance and effect size) findings of cingulum microstructure abnormalities are targeted along key segments of the bundle. These regions correspond to the connecting overlying cortices as they network through the cingulum to the wider limbic system and beyond (Table 3, Figure 4). Using a similar cohort, our group has previously found white matter differences in children and young adolescent with PEs using similar tractography techniques, particularly in tracts connecting frontotemporal regions, including the arcuate fasciculus (subcomponent of the superior longitudinal fasciculi) (Dooley et al., 2020) and putamen white matter (O’Hanlon et al., 2015). The differences were localised to projections of the right inferior fronto‐occipital fasciculus (reduced RD along 32% and increased FA along 16% of the tract) and left arcuate fasciculus (reduced RD along 39% of the tract). Our study found no difference in examining each cingulum section but uncovered large variations at the four section levels. Although our group established reduced RD as a shared finding in the left arcuate, inferior fronto‐occipital and uncinate fasciculi, we only found reduced RD in the left rostral retrosplenial and right subgenual areas of the cingulum. Our analysis established that FA levels differed depending on location with increased FA in the right rostral subgenual, left body and left retrosplenial subsegments and reduced FA in the right caudal‐mid subgenual, right body, left caudal retrosplenial and left parahippocampal subsegments. Due to its multiple spatially localised functions along its length, the cingulum is a more complex structure compared with the tracts examined by Dooley and O'Hanlon. Diverging diffusion metrics found in different white matter tracts of young individuals with PEs suggest that their broad use as vulnerability markers across brain areas may be questionable.

### 4.6 Strengths and limitations

This is the first study examining the cingulum using both a sectional and along tract analysis in young adolescents with PEs. Study strengths include a prospective cohort of a well‐characterised and matched community‐based sample of treatment/drug naïve adolescents. Of note, these robust findings were found in non‐clinical, medically well adolescents, who had not presented to their local family doctor or psychiatric services. High angular diffusion imaging (61 collinear directions), robust diffusion pre‐processing techniques and CSD tract reconstruction are also strengths of this study. Section reconstruction validity was maximised using a previously validated protocol, attaining a high interrater reliability coefficient (>.9) with third rater checks. All analyses were covaried for age, sex and eTIV, factors known to influence white matter metrics (Hsu et al., 2008). Due to the high number of comparisons, both Bonferroni and FDR were calculated for the along tract analysis with the strictest *p*‐value correction used as the new significance threshold (*p* < .00013) (see Supporting Information). We recognise limitations of this study including the relatively small sample size (*N* = 50 participants), requiring replication in a larger sample with international external replication and validation, as well as the documented limitations of diffusion tractography (Mori & Aggarwal, 2014). Results are also complicated and contextualised by the fact that the adolescent cingulum is still developing and continues to develop past the typical age of diagnosis of psychotic disorders (Kochunov et al., 2016).

## 5 CONCLUSION

This diffusion tractography study examines the cingulum of 25 adolescents with PEs who had not presented to services and 25 matched healthy controls. No differences were found when the cingulum was examined as four discrete anatomically consistent sections. However, along‐tract analysis of each section found considerable diffusion differences along continuous subsegments of the left retrosplenial and right subgenual sections of the bundle. These microanatomical changes in young people with PEs confirm, for the first time, a structural white matter basis for left‐sided medial prefrontal and caudal anterior cingulate cortical hyperfunction with right‐sided anterior cingulate and retrosplenial cortical hypofunction that can also be found in the broader psychosis phenotype. Our findings, in a community‐based cohort, also suggest that limbic connectivity difficulties, through a mechanism of cingulum abnormalities, may be common in a sub‐clinical cohort of young adolescents who experience PEs.

## CONFLICT OF INTEREST

The authors have no conflicts of interest to disclose.

## AUTHOR CONTRIBUTIONS

DWR and EOH were involved in recruitment, protocol design, data analysis and manuscript preparation. ER was involved in protocol design, tractography, data analysis and manuscript preparation. TB and LS performed tractography and were involved in manuscript preparations. AN performed statistical analysis. IK, AON, AN, AZ and MC were involved in the validation, writing and editing of the manuscript. MC was the principal investigator and was involved in manuscript preparation.

### PEER REVIEW

The peer review history for this article is available at https://publons.com/publon/10.1111/ejn.15806.

## Supporting information


**Table S1.** Supporting Information

## Data Availability

The data that support the findings of this study are available from the corresponding author upon reasonable request.

## References

[ejn15806-bib-0001] Abdul‐Rahman, M. F. , Qiu, A. , & Sim, K. (2011). Regionally specific white matter disruptions of fornix and cingulum in schizophrenia. PLoS ONE, 6(4), e18652. 10.1371/journal.pone.0018652 21533181 PMC3077390

[ejn15806-bib-0002] Aleksandrowicz, A. , Hagenmuller, F. , Haker, H. , Heekeren, K. , Theodoridou, A. , Walitza, S. , Ehlis, A. C. , Fallgatter, A. , Rössler, W. , & Kawohl, W. (2020). Frontal brain activity in individuals at risk for schizophrenic psychosis and bipolar disorder during the emotional Stroop task–an fNIRS study. NeuroImage: Clinical, 26, 102232. 10.1016/j.nicl.2020.102232 32272372 PMC7139160

[ejn15806-bib-0003] Alexander, A. L. , Hurley, S. A. , Samsonov, A. A. , Adluru, N. , Hosseinbor, A. P. , Mossahebi, P. , Tromp, D. P. M. , Zakszewski, E. , & Field, A. S. (2011). Characterization of cerebral white matter properties using quantitative magnetic resonance imaging stains. Brain Connectivity, 1(6), 423–446. 10.1089/brain.2011.0071 22432902 PMC3360545

[ejn15806-bib-0004] American Psychiatric Association . (2013). Diagnostic and statistical manual of mental disorders (DSM‐5®). American Psychiatric Pub.

[ejn15806-bib-0005] Andrade, L. , & Wang, Y. (2012). Prevalence of psychotic symptoms in the general population varies across 52 countries. Evidence‐Based Mental Health, 15(4), 105. 10.1136/ebmental-2012-100849 22815316

[ejn15806-bib-0006] Andreou, C. , & Borgwardt, S. (2020). Structural and functional imaging markers for susceptibility to psychosis. Molecular Psychiatry, 25(11), 2773–2785. 10.1038/s41380-020-0679-7 32066828 PMC7577836

[ejn15806-bib-0007] Armando, M. , Nelson, B. , Yung, A. R. , Ross, M. , Birchwood, M. , Girardi, P. , & Nastro, P. F. (2010). Psychotic‐like experiences and correlation with distress and depressive symptoms in a community sample of adolescents and young adults. Schizophrenia Research, 119(1–3), 258–265. 10.1016/j.schres.2010.03.001 20347272

[ejn15806-bib-0008] Bennett, J. C. , Surkan, P. J. , Moulton, L. H. , Fombonne, E. , & Melchior, M. (2019). Childhood social isolation and psychotic experiences in young adulthood: A community based study. European Child & Adolescent Psychiatry, 29, 1–8. 10.1007/s00787-019-01417-2 31595438

[ejn15806-bib-0009] Bersani, F. S. , Minichino, A. , Fojanesi, M. , Gallo, M. , Maglio, G. , Valeriani, G. , Biondi, M. , & Fitzgerald, P. B. (2014). Cingulate cortex in schizophrenia: Its relation with negative symptoms and psychotic onset. A review study. European Review for Medical and Pharmacological Sciences, 18(22), 3354–3367. 25491609

[ejn15806-bib-0010] Blanchard, M. M. , Jacobson, S. , Clarke, M. C. , Connor, D. , Kelleher, I. , Garavan, H. , Harley, M. , & Cannon, M. (2010). Language, motor and speed of processing deficits in adolescents with subclinical psychotic symptoms. Schizophrenia Research, 123(1), 71–76. 10.1016/j.schres.2010.05.028 20580205

[ejn15806-bib-0082] Bluhm, R. L. , Miller, J. , Lanius, R. A. , Osuch, E. A. , Boksman, K. , Neufeld, R. W. , Théberge, J. , Schaefer, B. , & Williamson, P. C. (2009). Retrosplenial cortex connectivity in schizophrenia. Psychiatry Research: Neuroimaging, 174(1), 17–23. 10.1016/j.pscychresns.2009.03.010 19783410

[ejn15806-bib-0011] Broome, M. R. , Matthiasson, P. , Fusar‐Poli, P. , Woolley, J. B. , Johns, L. C. , Tabraham, P. , Bramon, E. , Valmaggia, L. , Williams, S. C. R. , Brammer, M. J. , Chitnis, X. , & McGuire, P. K. (2009). Neural correlates of executive function and working memory in the ‘at‐risk mental state’. The British Journal of Psychiatry, 194(1), 25–33. 10.1192/bjp.bp.107.046789 19118321

[ejn15806-bib-0012] Bubb, E. J. , Metzler‐Baddeley, C. , & Aggleton, J. P. (2018). The cingulum bundle: Anatomy, function, and dysfunction. Neuroscience and Biobehavioral Reviews, 92, 104–127. 10.1016/j.neubiorev.2018.05.008 29753752 PMC6090091

[ejn15806-bib-0013] Calvo, A. , Roddy, D. W. , Coughlan, H. , Kelleher, I. , Healy, C. , Harley, M. , Clarke, M. , Leemans, A. , Frodl, T. , O’Hanlon, E. , & Cannon, M. (2020). Reduced hippocampal volume in adolescents with psychotic experiences: A longitudinal population‐based study. PLoS ONE, 15(6), e0233670. 10.1371/journal.pone.0233670 32492020 PMC7269246

[ejn15806-bib-0014] Carey, E. , Dooley, N. , Gillan, D. , Healy, C. , Coughlan, H. , Clarke, M. , Kelleher, I. , & Cannon, M. (2019). Fine motor skill and processing speed deficits in young people with psychotic experiences: A longitudinal study. Schizophrenia Research, 204, 127–132. 10.1016/j.schres.2018.08.014 30174253

[ejn15806-bib-0015] Carey, E. , Gillan, D. , Healy, C. , Dooley, N. , Campbell, D. , McGrane, J. , O'Neill, A. , Coughlan, H. , Clarke, M. , Kelleher, I. , & Cannon, M. (2020). Early adult mental health, functional and neuropsychological outcomes of young people who have reported psychotic experiences: A 10‐year longitudinal study. Psychological Medicine, 51(11), 1–9. 10.1017/S0033291720000616 32216843

[ejn15806-bib-0016] Colby, J. B. , Soderberg, L. , Lebel, C. , Dinov, I. D. , Thompson, P. M. , & Sowell, E. R. (2012). Along‐tract statistics allow for enhanced tractography analysis. NeuroImage, 59(4), 3227–3242. 10.1016/j.neuroimage.2011.11.004S1053-8119(11)01283-3[pii] 22094644 PMC3288584

[ejn15806-bib-0017] Coughlan, H. , Walton‐Ball, E. , Carey, E. , Healy, C. , O'Regan‐Murphy, G. , Uidhir, A. N. , Clarke, M. C. , & Cannon, M. (2021). Self‐reported interpersonal and educational/vocational difficulties in young adults with a history of transient psychotic experiences: Findings from a population‐based study. BMC Psychiatry, 21(1), 1–11. 10.1186/s12888-020-03022-z 33430829 PMC7802220

[ejn15806-bib-0019] Dooley, N. , O'Hanlon, E. , Healy, C. , Adair, A. , McCandless, C. , Coppinger, D. , Kelleher, I. , Clarke, M. , Leemans, A. , Frodl, T. , & Cannon, M. (2020). Psychotic experiences in childhood are associated with increased structural integrity of the left arcuate fasciculus—A population‐based case‐control study. Schizophrenia Research, 215, 378–384. 10.1016/j.schres.2019.08.022 31495700

[ejn15806-bib-0020] Epstein, K. A. , Cullen, K. R. , Mueller, B. A. , Robinson, P. , Lee, S. , & Kumra, S. (2014). White matter abnormalities and cognitive impairment in early‐onset schizophrenia‐spectrum disorders. Journal of the American Academy of Child & Adolescent Psychiatry, 53(3), 362–372.e2. 10.1016/j.jaac.2013.12.007 24565363 PMC3977613

[ejn15806-bib-0021] Falkenberg, I. , Chaddock, C. , Murray, R. M. , McDonald, C. , Modinos, G. , Bramon, E. , Walshe, M. , Broome, M. , McGuire, P. , & Allen, P. (2015). Failure to deactivate medial prefrontal cortex in people at high risk for psychosis. European Psychiatry, 30(5), 633–640. 10.1016/j.eurpsy.2015.03.003 25841662

[ejn15806-bib-0022] Fitzsimmons, J. , Rosa, P. , Sydnor, V. J. , Reid, B. E. , Makris, N. , Goldstein, J. M. , Mesholam‐Gately, R. I. , Woodberry, K. , Wojcik, J. , McCarley, R. W. , Seidman, L. J. , Shenton, M. E. , & Kubicki, M. (2020). Cingulum bundle abnormalities and risk for schizophrenia. Schizophrenia Research, 215, 385–391. 10.1016/j.schres.2019.08.017 31477373

[ejn15806-bib-0023] Frangou, S. , Haldane, M. , Roddy, D. , & Kumari, V. (2005). Evidence for deficit in tasks of ventral, but not dorsal, prefrontal executive function as an Endophenotypic marker for bipolar disorder. Biological Psychiatry, 58(10), 838–839. 10.1016/j.biopsych.2005.05.020 16043135

[ejn15806-bib-0024] Fujiwara, H. , Namiki, C. , Hirao, K. , Miyata, J. , Shimizu, M. , Fukuyama, H. , Sawamoto, N. , Hayashi, T. , & Murai, T. (2007). Anterior and posterior cingulum abnormalities and their association with psychopathology in schizophrenia: A diffusion tensor imaging study. Schizophrenia Research, 95(1–3), 215–222. 10.1016/j.schres.2007.05.044 17664062

[ejn15806-bib-0025] Fusar‐Poli, P. , Howes, O. D. , Allen, P. , Broome, M. , Valli, I. , Asselin, M.‐C. , Grasby, P. M. , & McGuire, P. K. (2010). Abnormal frontostriatal interactions in people with prodromal signs of psychosis: A multimodal imaging study. Archives of General Psychiatry, 67(7), 683–691. 10.1001/archgenpsychiatry.2010.77 20603449

[ejn15806-bib-0026] Garavan, H. , Ross, T. , & Stein, E. (1999). Right hemispheric dominance of inhibitory control: An event‐related functional MRI study. Proceedings of the National Academy of Sciences, 96(14), 8301–8306. 10.1073/pnas.96.14.8301 PMC22229 10393989

[ejn15806-bib-0076] Haupt, S. , Axmacher, N. , Cohen, M. X. , Elger, C. E. , & Fell, J. (2009). Activation of the caudal anterior cingulate cortex due to task‐related interference in an auditory Stroop paradigm. Human Brain Mapping, 30(9), 3043–3056. 19180558 10.1002/hbm.20731 PMC6870781

[ejn15806-bib-0028] Hazlett, E. A. , Goldstein, K. E. , Tajima‐Pozo, K. , Speidel, E. R. , Zelmanova, Y. , Entis, J. J. , Silverman, J. M. , New, A. S. , Koenigsberg, H. W. , Haznedar, M. M. , Byne, W. , & Siever, L. J. (2011). Cingulate and temporal lobe fractional anisotropy in schizotypal personality disorder. NeuroImage, 55(3), 900–908. 10.1016/j.neuroimage.2010.12.082 21223999 PMC3262398

[ejn15806-bib-0029] Healy, C. , Brannigan, R. , Dooley, N. , Coughlan, H. , Clarke, M. , Kelleher, I. , & Cannon, M. (2019). Childhood and adolescent psychotic experiences and risk of mental disorder: A systematic review and meta‐analysis. Psychological Medicine, 49(10), 1589–1599. 10.1017/S0033291719000485 31088578

[ejn15806-bib-0030] Hegarty, C. E. , Jolles, D. D. , Mennigen, E. , Jalbrzikowski, M. , Bearden, C. E. , & Karlsgodt, K. H. (2019). Disruptions in white matter maturation and mediation of cognitive development in youths on the psychosis spectrum. Biological Psychiatry: Cognitive Neuroscience and Neuroimaging, 4(5), 423–433. 10.1016/j.bpsc.2018.12.008 30745004 PMC6941904

[ejn15806-bib-0031] Hoptman, M. J. , Nierenberg, J. , Bertisch, H. C. , Catalano, D. , Ardekani, B. A. , Branch, C. A. , & DeLisi, L. E. (2008). A DTI study of white matter microstructure in individuals at high genetic risk for schizophrenia. Schizophrenia Research, 106(2–3), 115–124. 10.1016/j.schres.2008.07.023 18804959

[ejn15806-bib-0075] Hugdahl, K. (2015). Auditory hallucinations: A review of the ERC “VOICE” project. World Journal of Psychiatry, 5(2), 193–209. 26110121 10.5498/wjp.v5.i2.193 PMC4473491

[ejn15806-bib-0032] Hsu, J. L. , Leemans, A. , Bai, C. H. , Lee, C. H. , Tsai, Y. F. , Chiu, H. C. , & Chen, W. H. (2008). Gender differences and age‐related white matter changes of the human brain: A diffusion tensor imaging study. NeuroImage, 39(2), 566–577. 10.1016/j.neuroimage.2007.09.017 17951075

[ejn15806-bib-0034] Jalbrzikowski, M. , Murty, V. P. , Stan, P. L. , Saifullan, J. , Simmonds, D. , Foran, W. , & Luna, B. (2018). Differentiating between clinical and behavioral phenotypes in first‐episode psychosis during maintenance of visuospatial working memory. Schizophrenia Research, 197, 357–364. 10.1016/j.schres.2017.11.012 29137828 PMC5948111

[ejn15806-bib-0035] Jeurissen, B. , Leemans, A. , Jones, D. K. , Tournier, J. D. , & Sijbers, J. (2011). Probabilistic fiber tracking using the residual bootstrap with constrained spherical deconvolution. Human Brain Mapping, 32(3), 461–479. 10.1002/hbm.21032 21319270 PMC6869960

[ejn15806-bib-0037] Jones, D. K. , Christiansen, K. F. , Chapman, R. J. , & Aggleton, J. P. (2013). Distinct subdivisions of the cingulum bundle revealed by diffusion MRI fibre tracking: Implications for neuropsychological investigations. Neuropsychologia, 51(1), 67–78. 10.1016/j.neuropsychologia.2012.11.018 23178227 PMC3611599

[ejn15806-bib-0038] Kates, W. R. , Olszewski, A. K. , Gnirke, M. H. , Kikinis, Z. , Nelson, J. , Antshel, K. M. , Fremont, W. , Radoeva, P. D. , Middleton, F. A. , Shenton, M. E. , & Coman, I. L. (2015). White matter microstructural abnormalities of the cingulum bundle in youths with 22q11. 2 deletion syndrome: Associations with medication, neuropsychological function, and prodromal symptoms of psychosis. Schizophrenia Research, 161(1), 76–84. 10.1016/j.schres.2014.07.010 25066496 PMC4277733

[ejn15806-bib-0039] Kelleher, I. , Connor, D. , Clarke, M. C. , Devlin, N. , Harley, M. , & Cannon, M. (2012). Prevalence of psychotic symptoms in childhood and adolescence: A systematic review and meta‐analysis of population‐based studies. Psychological Medicine, 42(9), 1857–1863. 10.1017/S0033291711002960 22225730

[ejn15806-bib-0040] Kelleher, I. , Corcoran, P. , Keeley, H. , Wigman, J. T. , Devlin, N. , Ramsay, H. , Wasserman, C. , Carli, V. , Sarchiapone, M. , Hoven, C. , Wasserman, D. , & Cannon, M. (2013). Psychotic symptoms and population risk for suicide attempt: A prospective cohort study. JAMA Psychiatry, 70(9), 940–948. 10.1001/jamapsychiatry.2013.140 23863946

[ejn15806-bib-0041] Kelleher, I. , Harley, M. , Murtagh, A. , & Cannon, M. (2011). Are screening instruments valid for psychotic‐like experiences? A validation study of screening questions for psychotic‐like experiences using in‐depth clinical interview. Schizophrenia Bulletin, 37(2), 362–369. 10.1093/schbul/sbp057 19542527 PMC3044617

[ejn15806-bib-0042] Kelleher, I. , Lynch, F. , Harley, M. , Molloy, C. , Roddy, S. , Fitzpatrick, C. , & Cannon, M. (2012). Psychotic symptoms in adolescence index risk for suicidal behavior: Findings from 2 population‐based case‐control clinical interview studies. Archives of General Psychiatry, 69(12), 1277–1283. 10.1001/archgenpsychiatry.2012.1641386064[pii] 23108974

[ejn15806-bib-0043] Kelleher, I. , Murtagh, A. , Molloy, C. , Roddy, S. , Clarke, M. C. , Harley, M. , & Cannon, M. (2011). Identification and characterization of prodromal risk syndromes in young adolescents in the community: A population‐based clinical interview study. Schizophrenia Bulletin, 38, 239–246. 10.1093/schbul/sbr164 22101962 PMC3283157

[ejn15806-bib-0044] Kelleher, I. , Wigman, J. T. , Harley, M. , O'Hanlon, E. , Coughlan, H. , Rawdon, C. , Murphy, J. , Power, E. , Higgins, N. M. , & Cannon, M. (2015). Psychotic experiences in the population: Association with functioning and mental distress. Schizophrenia Research, 165(1), 9–14. 10.1016/j.schres.2015.03.020 25868930

[ejn15806-bib-0045] Kochunov, P. , Ganjgahi, H. , Winkler, A. , Kelly, S. , Shukla, D. K. , du, X. , Jahanshad, N. , Rowland, L. , Sampath, H. , Patel, B. , O'Donnell, P. , Xie, Z. , Paciga, S. A. , Schubert, C. R. , Chen, J. , Zhang, G. , Thompson, P. M. , Nichols, T. E. , & Hong, L. E. (2016). Heterochronicity of white matter development and aging explains regional patient control differences in schizophrenia. Human Brain Mapping, 37(12), 4673–4688. 10.1002/hbm.23336 27477775 PMC5118078

[ejn15806-bib-0046] Kubicki, M. , Westin, C.‐F. , Nestor, P. G. , Wible, C. G. , Frumin, M. , Maier, S. E. , Kikinis, R. , Jolesz, F. A. , McCarley, R. W. , & Shenton, M. E. (2003). Cingulate fasciculus integrity disruption in schizophrenia: A magnetic resonance diffusion tensor imaging study. Biological Psychiatry, 54(11), 1171–1180. 10.1016/S0006-3223(03)00419-0 14643084 PMC2806222

[ejn15806-bib-0077] Lahti, A. C., Weiler, M. A. , Holcomb, H. H. , Tamminga, C. A. , Carpenter, W. T. , & McMahon, R. (2006). Correlations between rCBF and symptoms in two independent cohorts of drug‐free patients with schizophrenia. Neuropsychopharmacology, 31(1), 221–230. 16123774 10.1038/sj.npp.1300837

[ejn15806-bib-0047] Leemans, A. , Jeurissen, B. , Sijbers, J. , & Jones, D. K. (2009). ExploreDTI: a graphical toolbox for processing, analyzing, and visualizing diffusion MR data. Proceedings of the International Society for Magnetic Resonance in Medicine, Honolulu, Hawaii.

[ejn15806-bib-0048] Leemans, A. , & Jones, D. K. (2009). The B‐matrix must be rotated when correcting for subject motion in DTI data. Magnetic Resonance in Medicine, 61(6), 1336–1349. 10.1002/mrm.21890 19319973

[ejn15806-bib-0049] Lord, L.‐D. , Allen, P. , Expert, P. , Howes, O. , Broome, M. , Lambiotte, R. , Fusar‐Poli, P. , Valli, I. , McGuire, P. , & Turkheimer, F. E. (2012). Functional brain networks before the onset of psychosis: A prospective fMRI study with graph theoretical analysis. NeuroImage: Clinical, 1(1), 91–98. 10.1016/j.nicl.2012.09.008 24179741 PMC3757719

[ejn15806-bib-0050] McGrath, J. , Johnson, K. , O'Hanlon, E. , Garavan, H. , Gallagher, L. , & Leemans, A. (2013). White matter and visuospatial processing in autism: A constrained spherical deconvolution tractography study. Autism Research, 6(5), 307–319. 10.1002/aur.1290 23509018

[ejn15806-bib-0081] Miller, A. M. , Vedder, L. C. , Law, L. M. , & Smith, D. M. (2014). Cues, context, and long‐term memory: the role of the retrosplenial cortex in spatial cognition. Frontiers in Human Neuroscience, 8, 586. 10.1016/j.neulet.2004.07.024 25140141 PMC4122222

[ejn15806-bib-0084] Mitelman, S. A. , Shihabuddin, L. , Brickman, A. M. , Hazlett, E. A. , & Buchsbaum, M. S. (2005). Volume of the cingulate and outcome in schizophrenia. Schizophrenia Research, 72(2‐3), 91–108. https://doi.org/10.1016/j.schres.2004.02.011 15560955 10.1016/j.schres.2004.02.011

[ejn15806-bib-0051] Mori, S. , & Aggarwal, M. (2014). In vivo magnetic resonance imaging of the human limbic white matter. Frontiers in Aging Neuroscience, 6, 321. 10.3389/fnagi.2014.00321 25505883 PMC4245919

[ejn15806-bib-0052] Nasa, A. , Gaughan, C. , Mahmoud, M. , Kelly, J. R. , Roman, E. , Levins, K. J. , Barry, D. , Frodl, T. , O'Hanlon, E. , O'Keane, V. , & Roddy, D. W. (2021). The human dorsal hippocampal commissure: Delineating connections across the midline using multi‐modal neuroimaging in major depressive disorder. Neuroimage: Reports, 1(4), 100062. 10.1016/j.ynirp.2021.100062 PMC12172789 40568434

[ejn15806-bib-0053] Nolan, M. , Roman, E. , Nasa, A. , Levins, K. J. , O'Hanlon, E. , O'Keane, V. , & Willian Roddy, D. (2020). Hippocampal and Amygdalar volume changes in major depressive disorder: A targeted review and focus on stress. Chronic Stress, 4, 2470547020944553. 10.1177/2470547020944553 33015518 PMC7513405

[ejn15806-bib-0054] O’Hanlon, E. , Leemans, A. , Kelleher, I. , Clarke, M. C. , Roddy, S. , Coughlan, H. , Harley, M. , Amico, F. , Hoscheit, M. J. , Tiedt, L. , Tabish, J. , McGettigan, A. , Frodl, T. , & Cannon, M. (2015). White matter differences among adolescents reporting psychotic experiences: A population‐based diffusion magnetic resonance imaging study. JAMA Psychiatry, 72(7), 668–677. 10.1001/jamapsychiatry.2015.0137 25923212

[ejn15806-bib-0055] O’Neill, A. , Carey, E. , Dooley, N. , Healy, C. , Coughlan, H. , Kelly, C. , Frodl, T. , O’Hanlon, E. , & Cannon, M. (2020). Multiple network Dysconnectivity in adolescents with psychotic experiences: A longitudinal population‐based study. Schizophrenia Bulletin, 46(6), 1608–1618. 10.1093/schbul/sbaa056 32614036 PMC7846103

[ejn15806-bib-0056] Oestreich, L. K. , Randeniya, R. , & Garrido, M. I. (2019). White matter connectivity reductions in the pre‐clinical continuum of psychosis: A connectome study. Human Brain Mapping, 40(2), 529–537. 10.1002/hbm.24392 30251761 PMC6865570

[ejn15806-bib-0057] Peters, B. D. , de Haan, L. , Dekker, N. , Blaas, J. , Becker, H. E. , Dingemans, P. M. , Akkerman, E. M. , Majoie, C. B. , van Amelsvoort, T. , den Heeten, G. J. , & Linszen, D. H. (2008). White matter fibertracking in first‐episode schizophrenia, schizoaffective patients and subjects at ultra‐high risk of psychosis. Neuropsychobiology, 58(1), 19–28. 10.1159/000154476 18781087

[ejn15806-bib-0058] Poulton, R. , Caspi, A. , Moffitt, T. E. , Cannon, M. , Murray, R. , & Harrington, H. (2000). Children's self‐reported psychotic symptoms and adult schizophreniform disorder: A 15‐year longitudinal study. Archives of General Psychiatry, 57(11), 1053–1058. 10.1001/archpsyc.57.11.1053 11074871

[ejn15806-bib-0059] Roddy, D. , & O'Keane, V. (2019). Cornu Ammonis changes are at the Core of hippocampal pathology in depression. Chronic Stress, 3, 2470547019849376. 10.1177/2470547019849376 32440594 PMC7219935

[ejn15806-bib-0060] Roddy, D. W. , Roman, E. , Rooney, S. , Andrews, S. , Farrell, C. , Doolin, K. , Levins, K. J. , Tozzi, L. , Tierney, P. , Barry, D. , Frodl, T. , O’Keane, V. , & O’Hanlon, E. (2018). Awakening neuropsychiatric research into the Stria medullaris: Development of a diffusion‐weighted imaging tractography protocol of this key limbic structure. Frontiers in Neuroanatomy, 12(39), 1–16. 10.3389/fnana.2018.00039 29867378 PMC5952041

[ejn15806-bib-0061] Shim, G. , Oh, J. S. , Jung, W. H. , Jang, J. H. , Choi, C.‐H. , Kim, E. , Park, H. Y. , Choi, J. S. , Jung, M. H. , & Kwon, J. S. (2010). Altered resting‐state connectivity in subjects at ultra‐high risk for psychosis: An fMRI study. Behavioral and Brain Functions, 6(1), 1–11. 10.1186/1744-9081-6-58 20932348 PMC2959003

[ejn15806-bib-0062] Stuss, D. T. , & Knight, R. T. (2013). Principles of Frontal Lobe Function. Oxford University Press. 10.1093/med/9780199837755.001.0001

[ejn15806-bib-0063] Takei, K. , Yamasue, H. , Abe, O. , Yamada, H. , Inoue, H. , Suga, M. , Muroi, M. , Sasaki, H. , Aoki, S. , & Kasai, K. (2009). Structural disruption of the dorsal cingulum bundle is associated with impaired Stroop performance in patients with schizophrenia. Schizophrenia Research, 114(1–3), 119–127. 10.1016/j.schres.2009.05.012 19505800

[ejn15806-bib-0064] Taylor, S. F. , Welsh, R. C. , Chen, A. C. , Velander, A. J. , & Liberzon, I. (2007). Medial frontal hyperactivity in reality distortion. Biological Psychiatry, 61(10), 1171–1178. 10.1016/j.biopsych.2006.11.029 17434455

[ejn15806-bib-0083] Tendolkar, I. , Weis, S. , Guddat, O. , Fernández, G. , Brockhaus‐Dumke, A. , Specht, K. , Klosterkötter, J. , Reul, J. , & Ruhrmann, S. (2004). Evidence for a dysfunctional retrosplenial cortex in patients with schizophrenia: a functional magnetic resonance imaging study with a semantic—perceptual contrast. Neuroscience Letters, 369(1), 4–8. 10.1016/j.neulet.2004.07.024 15380297

[ejn15806-bib-0065] van Os, J. , Linscott, R. J. , Myin‐Germeys, I. , Delespaul, P. , & Krabbendam, L. (2009). A systematic review and meta‐analysis of the psychosis continuum: Evidence for a psychosis proneness‐persistence‐impairment model of psychotic disorder. Psychological Medicine, 39(2), 179–195. 10.1017/S0033291708003814 18606047

[ejn15806-bib-0080] Vann, S. D. , Aggleton, J. P. , & Maguire, E. A. (2009). What does the retrosplenial cortex do? Nature Reviews Neuroscience, 10(11), 792–802. 10.1038/nrn2733 19812579

[ejn15806-bib-0066] Vogt, B. A. , & Paxinos, G. (2014). Cytoarchitecture of mouse and rat cingulate cortex with human homologies. Brain Structure & Function, 219(1), 185–192. 10.1007/s00429-012-0493-3 23229151

[ejn15806-bib-0067] Weininger, J. K. , Roman, E. , Tierney, P. , Barry, D. , Gallagher, H. , Murphy, P. , Levins, K. J. , O’Keane, V. , O'Hanlon, E. , & Roddy, D. W. (2019). Papez's forgotten tract: 80 years of unreconciled findings concerning the thalamocingulate tract. Frontiers in Neuroanatomy, 13(14), 1–11. 10.3389/fnana.2019.00014 30833890 PMC6388660

[ejn15806-bib-0068] Whitfield‐Gabrieli, S. , Thermenos, H. W. , Milanovic, S. , Tsuang, M. T. , Faraone, S. V. , McCarley, R. W. , Shenton, M. E. , Green, A. I. , Nieto‐Castanon, A. , LaViolette, P. , Wojcik, J. , Gabrieli, J. D. E. , & Seidman, L. J. (2009). Hyperactivity and hyperconnectivity of the default network in schizophrenia and in first‐degree relatives of persons with schizophrenia. Proceedings of the National Academy of Sciences, 106(4), 1279–1284. 10.1073/pnas.0809141106 PMC2633557 19164577

[ejn15806-bib-0070] Whitford, T. J. , Lee, S. W. , Oh, J. S. , de Luis‐Garcia, R. , Savadjiev, P. , Alvarado, J. L. , Westin, C. F. , Niznikiewicz, M. , Nestor, P. G. , McCarley, R. W. , Kubicki, M. , & Shenton, M. E. (2014). Localized abnormalities in the cingulum bundle in patients with schizophrenia: A diffusion tensor tractography study. Neuroimage Clin, 5, 93–99. 10.1016/j.nicl.2014.06.003 25003032 PMC4081981

[ejn15806-bib-0071] Wilkins, B. , Lee, N. , Gajawelli, N. , Law, M. , & Lepore, N. (2015). Fiber estimation and tractography in diffusion MRI: Development of simulated brain images and comparison of multi‐fiber analysis methods at clinical b‐values. NeuroImage, 109, 341–356. 10.1016/j.neuroimage.2014.12.060 25555998 PMC4600612

[ejn15806-bib-0073] Zhou, Y. , Liu, J. , Driesen, N. , Womer, F. , Chen, K. , Wang, Y. , Jiang, X. , Zhou, Q. , Bai, C. , Wang, D. , Tang, Y. , & Wang, F. (2017). White matter integrity in genetic high‐risk individuals and first‐episode schizophrenia patients: Similarities and disassociations. BioMed Research International, 2017, 1–9. 10.1155/2017/3107845 PMC5376415 28401151

